# Comparison of learning curves for laparoendoscopic single-site myomectomy performed by 2 surgeons

**DOI:** 10.1097/MD.0000000000029830

**Published:** 2022-06-30

**Authors:** Yuanyuan Lu, Fan Yang, Longxia Tong, Ying Zheng

**Affiliations:** a Department of Gynecologic Oncology, West China Second Hospital, Sichuan University, Chengdu, Sichuan, China; b Key Laboratory of Obstetrics and Gynecologic and Pediatric Diseases and Birth Defects of Ministry of Education, West China Second Hospital, Sichuan University, Chengdu, Sichuan, People’s Republic of China.

**Keywords:** cumulative sum analysis, laparoendoscopic single-site surgery, learning curve, myomectomy

## Abstract

We aimed to compare the learning curves of 2 surgeons with different endoscopic bases when performing laparoendoscopic single-site myomectomy (LESS-M).

We retrospectively analyzed and compared 2 groups of patients who underwent LESS-M performed by 2 surgeons with different bases in multi-port laparoscopic surgery (MLS) from October 2019 to December 2020 at West China Second Hospital of Sichuan University. Patients’ characteristics and related surgical indicators were compared, and surgeons’ learning curves were analyzed using a cumulative sum analysis.

All of the patients completed LESS-M without converting to MLS or laparotomy, despite Surgeon A being MLS-unqualified and Surgeon B being MLS-qualified. There were no significant differences in patients’ characteristics or surgical indicators between the 2 groups (*P* > 0.05 for all). Surgeons A and B crossed the learning curve after 21 and 18 cases, respectively.

LESS-M is safe and feasible. Approximately 20 cases are required for surgeons to achieve LESS-M proficiency, and surgeons without MLS experience can still master LESS-M.

## 1. Introduction

With the development of minimally invasive surgery and the accumulation of surgical experience, laparoendoscopic single-site surgery (LESS) is developing rapidly. Compared with laparotomy and multiport laparoscopic surgery (MLS), LESS inflicts less physical trauma and facilitates a quicker recovery. Many studies have confirmed the safety and feasibility of LESS in patients with gynecological diseases.^[1–5]^ However, LESS is challenging for surgeons due to the narrow operating space, loss of triangulation, frequent clashing of instruments, and challenging suturing. Previously, LESS required surgeons to be skilled in MLS; however, with the advancement of LESS, surgeons without MLS experience can now master this technology. We conducted this retrospective cohort study to analyze the learning curves for laparoendoscopic single-site myomectomy (LESS-M) of 2 LESS novices (MLS-unqualified Surgeon A and MLS-qualified Surgeon B) using a cumulative sum (CUSUM) analysis.

## 2. Material and methods

We retrospectively reviewed the data of 63 patients who underwent LESS-M performed by 2 surgeons from October 2019 to December 2020 at West China Second Hospital of Sichuan University. Among them, 33 patients were conducted by surgeon A, and the remaining 30 patients were operated on by surgeon B. The surgeons were 2 attending physicians (MLS-unqualified Surgeon A and MLS-qualified Surgeon B). Surgeon A had assisted in nearly 100 MLS but had no experience in independent MLS, while surgeon B had independently conducted over 200 cases of benign gynecological MLS. These 2 surgeons had rich experience as LESS assistants but had no first-hand experience performing LESS. None of the patients had surgical contraindications, and the 2 surgical teams were relatively fixed. Data were collected from each surgeon first completion of LESS-M. This study was approved by the Institutional Ethics Committee of West China Second Hospital, Sichuan University.

### 2.1. Surgical methods

**Fig. 1.**
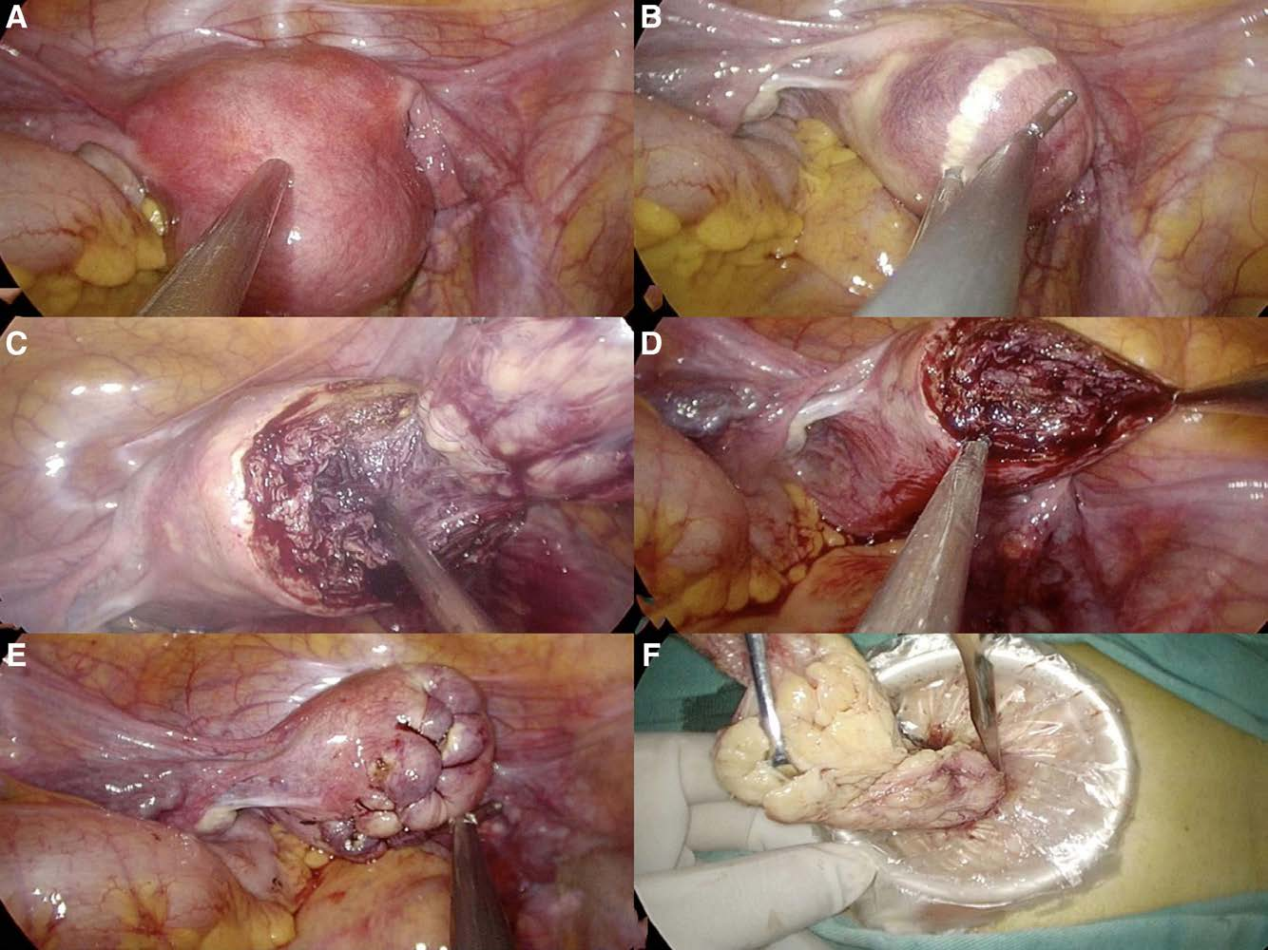
Key procedure. (A) Inject the diluted Pituitrin; (B) Mark the incision; (C) Remove the myoma; (D) Hemostasis; (E) Baseball stitching method with absorbable barb suture; (F) Remove the myoma with an in-bag cold knife.

First, an umbilical incision (within 2 cm) was made. A LESS port was placed after entering the abdomen layer by layer. Next, the location and size of uterine myoma were explored, and Pituitrin 6 U diluted in 20 ml physiological saline was injected into the uterine body to reduce bleeding (Fig. 1A). Then, a wedge-shaped incision was marked with a bipolar electrotome on the serosal surface of the uterus, and unipolar electrocoagulation was used to cut the serosal surface completely, exposing the myoma (Fig. 1B)). Multi-toothed forceps were used to grasp and remove the myoma (Fig. 1C). After coagulation of active vascular hemorrhage, an absorbable barb suture (SXPP1A400, Ethicon; Johnson & Johnson Medical Ltd., Guaynabo, Puerto Rico) was used to close the wound with the baseball suture technique, without leaving a dead cavity (Fig. 1D, E). In cases of multiple myoma, we followed the principle of largest first, followed by smallest, and then from shallow to deep, using the smallest possible incision. The removed myoma was placed in a simple specimen bag and then rotated with a cold knife and removed through the umbilical incision (Fig. 1F). Finally, the umbilical incision was sutured using Zheng Anchoring Suturing Technology.^[6,7]^ General anesthesia with endotracheal intubation was conducted in all patients in the Trendelenburg position, and the average pneumoperitoneal pressure was 14 mm Hg (1.862 kPa).

### 2.2. Data collection and definitions

Data were consulted and collected by 1 doctor. Collected data were as follows: (1) patients’ characteristics: age, body mass index (BMI), history of abdominal surgery, and preoperative hemoglobin (Hb); (2) surgical situation: operation time (OT), the volume of intraoperative blood loss, the decline in Hb, length of postoperative hospitalization, myoma number, maximal myoma diameter, and myoma location. OT refers to the time from the beginning of the skin incision to completion of skin suture. The volume of intraoperative blood loss was defined as aspirator suction volume. Length of postoperative hospitalization refers to the number of days from the first day after surgery to the day of discharge. The decline in Hb was the difference between the preoperative Hb value and the Hb value on the morning after surgery. Maximal myoma diameter refers to the maximum diameter of the removed myoma detected intraoperatively. Myoma location refers to the location from which the largest myoma was removed and was described as anterior, posterior, lateral, or fundal. Complications mainly included a decline in Hb requiring blood transfusion, subcutaneous emphysema, puncture wound, and adjacent organ injury, and complications were graded according to the Clavien–Dindo classification system.^[8]^

### 2.3. CUSUM analysis

The CUSUM formula [CUSUM (n) = (OTn − OTmean) + CUSUM (n−1)] was used to analyze the learning curves of surgeons, where n refers to the case number of each patient, OTn is the operation time of each patient, and OTmean is the mean operation time of each group. SPSS 26.0 software was used for curve fitting. A *P* value of <0.05 indicated successful fitting, and determination coefficient *R^2^* determined the goodness of fit. When the slope (k) was negative, the surgeon was deemed to have crossed the learning curve.

### 2.4. Statistical analysis

SPSS 19.0 statistical software (IBM, Armonk, NY) was used for all statistical analyses. Measurement data with a normal distribution are expressed as (^–^*x* ± *s*), and the *t*-test was used to identify significant differences. Non-normally distributed data are presented as medians, and the Mann–Whitney *U* test was performed to identify significant differences. Enumeration data are expressed as n (%) and were compared using the *χ2* test or Fisher exact probability method. A *P* value of <0.05 was considered statistically significant.

## 3. Results

### 3.1. Patient characteristics

There were no statistically significant differences in age, BMI, history of abdominal surgery, and preoperative Hb value between the 2 groups (*P* > 0.05 for all) (Table 1).

**Table 1:** Characteristics of patients.

Indicators	Group A (n = 33)	Group B (n = 30)	Test value	*P*
Age (years)	39 (32.5–42.0)	39.5 (33–41.3)	*Z* = −0.331	.740
BMI (kg/m^2^)	21.6 ± 2.9	22.3 ± 2.1	*t* = −1.133	.262
Preoperative Hb value (g/L)	134 (114.5–141.5)	128.5 (114–137.3)	*Z* = 0.847	.397
History of abdominal surgery (times)	0 (0–0.5)	0 (0–1)	*Z* = −1.165	.244

BMI = body mass index, Hb = hemoglobin.

### 3.2. Surgical situation and complications

All patients underwent successful surgery without converting to MLS or laparotomy. There were no significant differences in OT, the volume of intraoperative blood loss, the decline in Hb value, length of postoperative hospital stay, myoma number, maximal myoma diameter, myoma location, or complication rate between the 2 groups (Table 2). Three patients in each group had a Hb value <70 g/L on the day after surgery, but all of these patients recovered after undergoing treatment (Clavien–Dindo classification grade II) (Table 3). There were no other perioperative complications in this study.

**Table 2:** Surgical situation and complications.

Indicators	Group A (n = 33)	Group B (n = 30)	Test value	*P*
OT (min)	110 (85–140)	117.5 (91.5–135)	*Z* = −0.110	.912
Intraoperative blood loss (ml)	50 (20–100)	50 (20–100)	*Z* = −0.226	.821
Hb decline value (g/L)	18.4 ± 8.4	17.7 ± 6.9	*t* = −0.340	.735
Postoperative hospital stay (days)	2 (2–3)	3 (2–3.3)	*Z* = −1.679	.093
Myoma number			*	.819
1	25 (75.8)	21 (70)		
2–5	7 (21.2)	7 (23.3)		
>5	1 (3)	2 (6.7)		
Maximal myoma diameter (cm)	7 (6–8)	6.5 (5–9.3)	*Z* = −0.056	.955
Myoma location [n (%)]			*	.512
Anterior	11 (33.3)	13 (43.4)		
Posterior	18 (54.5)	12 (40)		
Lateral	3 (9.1)	2 (6.7)		
Fundal	1 (3)	3 (10)		
Complications	3 (9.1)	3 (10.0)	*	1.000

* Fisher exact probability method was used; so there was no *χ^2^* value.

OT = operation time, Hb = hemoglobin.

**Table 3:** Complications of patients.

	Preoperative Hb (g/L)	Postoperative Hb (g/L)	Corresponding treatment
Patient 1	74	56	Blood transfusion 4 U after surgery
Patient 2	86	63	Blood transfusion 3 U after surgery
Patient 3	90	69	Intravenous iron supplementation*
Patient 4	80	69	Blood transfusion 3 U during surgery and 3 U after surgery†
Patient 5	93	64	Blood transfusion 3 U after surgery
Patient 6	97	69	Intravenous iron supplementation*

* Indicates that the patient rejected blood transfusion.

† Indicates that the patient lost 400 ml of blood during surgery, so 3 U of blood was transfused during surgery.

Hb = hemoglobin.

### 3.3. Learning curves

**Fig. 2.**
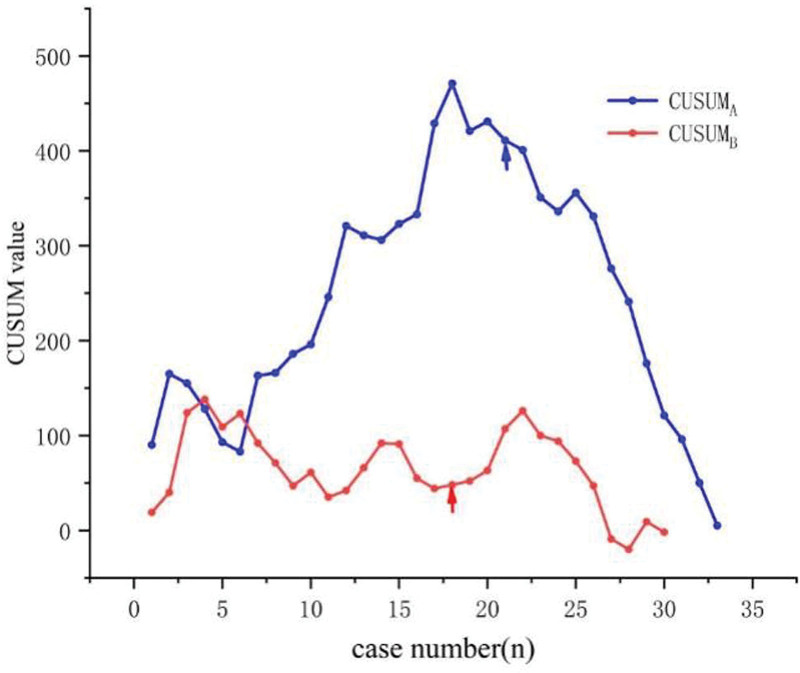
Learning curves using the CUSUM method.

The learning curves of both surgeons are shown in Figure 2. The optimal learning curve fitting formula in group A was CUSUM_A_ = −0.073X^3^ + 2.376X^2^ − 3.575X + 103.094 (*R^2^* = 0.920, *P* < 0.05), and the optimal curve fitting formula in group B was CUSUM_B_ = −0.017X^3^ + 0.624X^2^ − 6.392X + 92.267 (*R^2^* = 0.303, *P* < 0.05). The partial slope (k) values of the 2 learning curves are shown in Table 4.

**Table 4:** Partial slope (k) values.

Case number	17	18	19	20	21
k_A_	13.918	11.005	7.654	3.865	−0.362
k_B_	0.085	−0.452	−1.091	−1.832	−2.675

## 4. Discussion

LESS is emerging in gynecology and has better cosmetic results, especially favored by women.^[9]^ Additionally, it can enter the abdomen layer by layer with straight sight, avoiding the damage of the first puncture site, excel in patients with a history of repeated abdominal surgery or abdominal cavity infection.^[10]^ It was confirmed that LESS-M has been safe and feasible in recent years.^[11–14]^

### 4.1. Comparison of safety, feasibility, and learning curve of LESS-M in 2 types of surgeons

The cultivation of surgical skills should be based on the premise of safety and feasibility. All cases completed LESS-M successfully, without converting to other approaches. Three patients in each group met blood transfusion indication (Clavien-Dindo II) and recovered after the treatment with blood transfusion or intravenous iron supplementation (Table 3). All of these patients removed myoma larger than 5 cm. Moreover, they all had mild-moderate preoperative anemia, considering that Hb decline is associated with large myoma having rich blood supply, affecting exposure of the surgical field, and poor tolerance of blood loss with anemia. There was no statistical significance in the complications rate (*P >* 0.05). Surgeons’ not having MLS experience would not increase the perioperative complication rate.

The learning curve is usually used to study the learning rules of surgical technologies, which is measured by the number of cases required for novices to master surgical techniques. Studies have reported the learning curve of LESS-M, among which Ma et al found that MLS-qualified surgeons reached the plateau earlier than those without MLS basics (27 cases vs 35 cases, *P* < 0.05), and the mean OT of the former (101.84 min) was shorter than that of the latter (118.98 min).^[15–17]^ However, the surgical modes of MLS and LESS are quite different. MLS is mainly operated by both hands simultaneously, with instruments moving left-right direction. By contrast, due to the lack of incision fulcrum and “operation triangle,” LESS surgical instruments are required to move parallelly in an anterior-posterior direction. Owing to these differences, the fixed operation habit of MLS may hinder the rapid mastery of LESS in practice. Experienced MLS surgeons need to change their original surgical habits and switch to another new surgical mode, while surgeons without MLS experience can learn and adapt to the specific surgical mode of LESS. In our study, surgeon B also reached the plateau before surgeon A, but the cutoff point of the learning curve of the 2 groups was similar (18 cases vs 21 cases), and there were no significant differences between the 2 groups in OT, intraoperative blood loss, postoperative Hb decline, and other operation-related indicators, as well as the postoperative complications rate. The results showed that LESS-M could be mastered quickly and safely by MLS-unqualified novices.

### 4.2. Advantages and tips of LESS-M

Result from the longer and extensible umbilical incision of LESS, myomas can be removed with in-bag cold knife, avoiding the use of cumbersome closed rotary cut bag,^[5,18]^ furthermore, avoiding the pelvic and abdominal implantation of occult sarcoma and disseminating uterine leiomyoma caused by breaking myoma with electric rotary excision instruments, conforming to tumor-free principle, especially suitable for myomectomy. LESS-M includes almost all of the basic laparoscopic operation skills, like cutting,^[19–21]^ hemostasis, suture, and knot, which is challenging to novices. Hence attention should be paid to these points: (1) LESS-M lacks assistants to expose, and the difficulty of special locations such as posterior and cervical myoma is relatively increased. On this occasion, uterine manipulation can be used to swing the uterine and expose the surgical field. Moreover, for patients who have no sexual life, abdominal wall anchoring sutures can also be used to expose the operation field. (2) Compared with other gynecological benign surgeries, myomectomy products more smoke. Besides, LESS has weaker smoke exhaustion. The automatic smoke exhaust system, which can automatically exhaust smoke at the same time as energy instrument excitation, may partially improve the smoke exhaust effect. (3) Wounding suture is essential to myomectomy, whereas conventional continuous locking suture has a distant stitch length, surgeons’ strong or wrong-direction string may cause muscular tears, needle oozing, or leave space. Baseball Stitching is more applied to LESS-M.^[22]^ It inserts a needle at the bottom of the myoma chamber to close the wound completely. Meanwhile, both sides of the myometrium of the incision can apply to decrease bleeding. Additionally, absorbable barb sutures can be used to reduce the difficulty and shorten the suture time without increasing the incidence of postoperative adhesions.^[7,23]^ Novices of LESS-M can choose Baseball Stitching with absorbable barb sutures. (4) Learning curve is not only related to surgeons’ ability, but patients’ characteristics as the history of abdominal surgery, BMI, myoma size, location, and myoma number. Novices should evaluate patients cautiously preoperatively, simple one first and then the difficult, to build up confidence and ensure safety.

There are some limitations of our study that should be noted. Our study only examined the learning curves of 2 surgeons. Thus, the results should be verified in further studies. And further follow-up is needed to study the long-term complications and patients’ satisfaction after LESS.

In conclusion, LESS has prominent advantages in myomectomy. Although LESS-M is more challenging than MLS and requires surgeons to experience several cases to master the technique, novices without MLS experience can still master it safely and feasibly.

#### Author contributions

Conceptualization: Yuanyuan Lu.

Data curation: Yuanyuan Lu, Fan Yang, Longxia Tong.

Investigation: Yuanyuan Lu, Fan Yang, Longxia Tong.

Methodology: Yuanyuan Lu.

Supervision: Ying Zheng.

Writing – original draft: Yuanyuan Lu.

Writing – review and editing: Ying Zheng.

#### Acknowledgment

The authors thank Ms Li Mingyue for her guidance on statistical analysis.
